# Ginsenoside Rb1 attenuates diabetic retinopathy in streptozotocin-induced diabetic rats^1^

**DOI:** 10.1590/s0102-8650201900201

**Published:** 2019-02-28

**Authors:** Changxia Dong, Peng Liu, Huaizhou Wang, Mei Dong, Guangxin Li, Yuanbin Li

**Affiliations:** I Master, Department of Ophthalmology, Yantai Yuhuangding Hospital, P.R. China. Acquisition of data, manuscript writing.; II Master, Department of Ophthalmology, Yantai Yuhuangding Hospital, P.R. China. Manuscript writing.; III Master, Department of Anesthesiology, Yantai Stomatological Hospital, P.R. China. Acquisition of data.; IV Master, Department of Ophthalmology, Yantai Yuhuangding Hospital, P.R. China. Analysis and interpretation of data.; V MD, Department of Ophthalmology, Yantai Yuhuangding Hospital, P.R. China. Conception and design of the study, critical revision, final approval.

**Keywords:** Ginsenosides, Diabetic Retinopathy, Glutathione, Rats

## Abstract

**Purpose:**

To investigated the effects of ginsenoside Rb1 on diabetic retinopathy in streptozotocin-induced diabetic rats.

**Methods:**

Diabetes was induced by a single intraperitoneal injection of streptozotocin (80 mg/kg) in male Wistar rats. Ginsenoside Rb1 (20, 40 mg/kg) was injected (i.p.) once a day for 4 weeks. Then, using fundus photography, the diameter and vascular permeability of retinal vessels were investigated. Retinal histopathology was undertaken. Contents of malondialdehyde (MDA) and glutathione (GSH) in retinas were assayed. Levels of nuclear factor erythroid 2-related factor 2 (Nrf2), glutathione cysteine ligase catalytic subunit (GCLC), and glutathione cysteine ligase modulatory subunit (GCLM) were measured.

**Results:**

Treatment with ginsenoside Rb1 attenuated the diabetes-induced increase in the diameter of retinal blood vessels. Ginsenoside Rb1 reduced extravasation of Evans Blue dye from retinal blood vessels. Ginsenoside Rb1 partially inhibited the increase in MDA content and decrease in GSH level in rat retinas. Nrf2 levels in the nuclei of retinal cells and expression of GCLC and GCLM were increased significantly in rats treated with ginsenoside Rb1.

**Conclusion:**

These findings suggest that ginsenoside Rb1 can attenuate diabetic retinopathy by regulating the antioxidative function in rat retinas.

## Introduction

 Diabetes mellitus is a metabolic disease that affects more than 170 million people worldwide. Despite a new generation of medications and advances in clinical treatments, the prevalence of diabetes mellitus has risen dramatically in recent decades. 

 Diabetic retinopathy (DR) is a microvascular complication of diabetes mellitus, and is one of the major causes of vision loss worldwide. It has been demonstrated that over one-third of patients with diabetes mellitus have signs of DR, and the increasing prevalence of diabetes mellitus suggests that many more people will suffer from DR in the future^1^. 

 DR is characterized by progressive damage to the retinal microvasculature. It can be classified into two types: non-proliferative and proliferative^2^. In non-proliferative DR, the intra-retinal microvasculature is associated with diabetic macular edema^3^. Proliferative DR is implicated in the formation and growth of new blood vessels in low-oxygen environments^4^. DR is also characterized by increased vascular permeability, which leads to fluid accumulation and retinal hemorrhage in the macula^5^. 

 Hyperglycemia promotes the formation of reactive oxygen species (ROS) by disturbing the pathways of glycolysis and the citric-acid cycle. Several of the factors that can contribute to DR pathogenesis are related to hyperglycemia-induced oxidative stress (i.e., the imbalance between the formation and elimination of ROS)^6^. Increased cytosolic ROS damage the inner membrane of the mitochondria, which results in increased superoxide levels, and further damage to the membrane proteins^7^. Nuclear factor erythroid 2-related factor 2 (Nrf2) regulates the basal and inducible expression of genes playing important role in maintaining the oxidative homeostasis by regulating multiple downstream antioxidants defense genes, including superoxide dismutase, glutathione reductase and glutathione peroxidase. In the retina of diabetes, the DNA binding activity of Nrf2 is decreased. In addition, the transcripts of glutathione cysteine ligase catalytic subunit (GCLC), glutathione cysteine ligase modulatory subunit (GCLM) and the level of glutathione (GSH) are also decreased^8^. There has been considerable interest in increasing antioxidant capacity via Nrf2 for the treatment of diabetic retinopathy.

*Panax ginseng* has been used since ancient times in China. Its use is based on the theory of traditional Chinese medicine and clinical experiences. Ginsenosides are the major pharmacologically active ingredients of *Panax ginseng*. They are responsible for most of the activities of *Panax ginseng*: antioxidation, anti-inflammation and anti-cancer. 

 To date, more than 40 ginsenoside compounds have been identified. Depending on their structures, ginsenosides are divided into three groups: panaxadiol (which includes Rb1, Rb2, Rb3, Rg3, Rc, Rd and Rh2), panaxatriol (Re, Rf, Rg1, Rg2, and Rh1) and oleanolic acid^9^. Ginsenoside Rb1 is one of the main bioactive components of *Panax ginseng*. In adipocytes, ginsenoside Rb1 reduces ROS generation and shows antioxidative activity through upregulation of superoxide dismutase (SOD) expression^10^. Ginsenoside Rb1 exhibits a neuroprotective property that has been implicated in activation of the Nrf2 signaling pathway, reduction of ROS levels, and improvement of the glutathione (GSH) system^11^. Ginsenoside Rb1 reduces ROS levels and increases the SOD activity in the skeletal muscles of older rats. The mechanism of action of the antioxidative property of ginsenoside Rb1 may involve the activation of the phosphoinositide 3-kinase (PI3K)/Akt pathway with subsequent nuclear translocation of Nrf2 and induction of antioxidant enzymes^12^. In dopaminergic cell cultures, ginsenoside Rb1 has been shown to augment cellular antioxidant defenses by enhancing the PI3K/Akt/Nrf2 signaling pathway, thereby protecting cells from oxidative stress^13^. Here, we investigated the effect of ginsenoside Rb1 on DR in streptozotocin (STZ)-induced diabetic rats. 

## Methods 

###  Materials 

 Ginsenoside Rb1, STZ, EBD, GSH and *o*-phthalaldehyde were purchased from Sigma-Aldrich (Saint Louis, MO, USA). Glucose analyzer and strips (Accu-Chek Glucotrend 2) were obtained from Roche Diagnostics (Mannheim, Germany). Rabbit anti-Nrf2, anti-GCLC, anti-GCLM and anti-Lamin B1 antibodies were purchased from Abcam (Cambridge, MA, USA). Rabbit anti-β-Actin antibody was from Cell Signaling Technologies (Danvers, MA, USA). Electrochemiluminescence detection reagents and a bicinchoninic acid (BCA) protein assay kit were from Beyotime Institute of Biotechnology (Shanghai, China).

###  Animals 

 Experiments were undertaken according to Guidelines for the Care and Use of Laboratory Animals (publication 86-23, revised in 1986; US National Institutes of Health, Bethesda, MD, USA) and approved by the Ethics Committee of Yantai Yuhuangding Hospital (Yantai, P.R. China).

 Wistar rats (230-260 g) were purchased from Beijing HFK Bioscience (Beijing, China). Animals were housed in diurnal lighting conditions (12 h/12 h) and allowed free access to food and water for 7 days before experimentation. 

###  Experimental protocols 

 Rats were divided randomly into four groups of 16: control, diabetes, ginsenoside Rb1 (20 mg/kg) and ginsenoside Rb1 (40 mg/kg). A model of diabetes mellitus was induced by a single intraperitoneal injection of STZ (80 mg/kg, dissolved in sodium citrate solution, 0.1 mmol/L, pH 4.5). Age-matched rats were injected with sodium citrate solution. After 72 h, levels of fasting blood glucose were assayed using a glucose analyzer. Animals with blood glucose levels >16.67 mmol/L were selected as diabetic rats. The latter were injected (i.p.) with ginsenoside Rb1 at 20 or 40 mg/kg once a day for 4 weeks. Four weeks later, the body weight and level of fasting blood glucose were determined. 

###  Fundus photography 

 Animals were anesthetized with isoflurane. Pupils were dilated by application of 1% tropicamide to the eye. Then, rats were placed beneath a retinal camera (TRC-50IX; Topcon Medical Systems, Tokyo, Japan). The lens was adjusted until a clear image was focused on the retina. The image from each rat was scanned and the mean diameter of retinal vessels obtained using Image-Pro Plus (Media Cybernetics, Silver Spring, MA, USA).

###  Estimation of retinal vascular permeability 

 EBD extravasation was used to determine vascular permeability. EBD was injected (45 mg/kg) through a tail vein. After allowing it to circulate for 1 h, rats were perfused *via* a transcardial approach with 100 mL of ice-cold phosphate-buffered saline (PBS). After euthanasia with an overdose of isoflurane, eyes were collected, frozen in liquid nitrogen, and stored at −80°C until analyses. Samples were homogenized in 0.5 mL of PBS, sonicated, and centrifuged (12,000 *g*, 30 min, 4°C). The supernatant was collected and, for each 200-μL sample, an equal amount of 50% trichloroacetic acid was added. Samples were incubated overnight at 4°C and then centrifuged (12,000 *g*, 30 min, 4°C). EBD content was measured by an enzyme-linked immunosorbent assay plate reader (Bio-Rad Laboratories, Hercules, CA, USA) at 610 nm and quantified according to a standard curve. 

###  Histopathology 

 After euthanasia with an overdose of isoflurane, rat eyes were harvested. Subsequently, paraformaldehyde (4%)-fixed, paraffin-embedded samples were cut into 4-µm sections, deparaffinized in xylene and rehydrated through a series of decreasing concentrations of ethanol. Sections were stained with hematoxylin and eosin. Tissues were observed under a light microscope (IX83; Olympus, Tokyo, Japan).

###  MDA and GSH assays 

 Rat eyes were homogenized in 4 volumes of 0.1 mol/L ice-cold PBS and then centrifuged (10,000 × *g*, 15 min, 4°C). The total protein in the supernatant was estimated by a BCA protein assay kit. MDA content in eye tissues was assayed according to the method described by Ohkawa and colleagues^14^. GSH was measured using an *o*-phthalaldehyde condensation reaction with GSH at pH 8.0. Absorbance values were read at an activation wavelength of 340 nm and emission wavelength of 420 nm^15^. 

###  Western blotting 

 The proteins within rat eyes were extracted. Then, the proteins (60 µg) were separated by sodium dodecyl sulfate-polyacrylamide gel electrophoresis. After blockade with 5% non-fat milk for 2 h, cell membranes were incubated overnight at 4°C with primary antibodies: rabbit anti-Nrf2 (1:1000 dilution), anti-GCLC (1:2000), anti-GCLM (1:1500), anti-Lamin B1 (0.1 μg/mL) or rabbit anti-β-Actin (1:1000). β-Actin served as the loading control. Cell membranes were processed with the respective horseradish peroxidase-labeled secondary antibody. Bands were visualized using electrochemiluminescence detection reagents. The relative density of protein was analyzed by Image J (San Diego, CA, USA).

###  Statistical analyses 

 Data are the mean ± SD. Statistical significance was determined using one-way ANOVA followed by the Tukey *post hoc* test. Statistical significance was defined as *P* < 0.05. 

## Results

###  Effect of ginsenoside Rb1 on body weight and blood glucose level 

**Figure 1:**
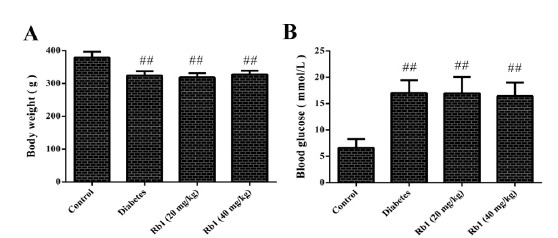
Effect of ginsenoside Rb1 on body weight and blood glucose level. **A**: Body weight; **B**: Blood glucose level; data are the mean ± SD, (n = 16). ##P < 0.01 compared with the control group.

 The body weight and blood glucose level of rats were recorded at the end of experimentation. Compared with the control group, rats in the diabetes group had a high blood glucose level (*P* < 0.01), accompanied by a low body weight (*P* < 0.01; Fig. 1). Compared with the diabetes group, treatment with ginsenoside Rb1 (20, 40 mg/kg body weight) had no effect on body weight or blood glucose level.

###  Effect of ginsenoside Rb1 on the diameter of retinal vessels and fundus photography 

**Figure 2:**
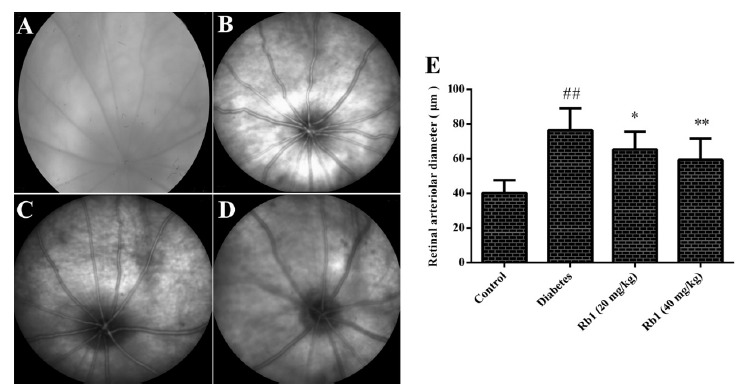
Effect of ginsenoside Rb1 on the diameter of retinal vessels and fundus photography. Representative images of fundus photography. **A**: Control group; **B**: Diabetes group; **C**: Ginsenoside Rb1 (20 mg/kg) group; **D**: Ginsenoside Rb1 (40 mg/kg) group; **E**: Diameter of retinal vessels. Data are the mean ± SD, (n = 16). ##P < 0.01 compared with the control group; *P < 0.05 or **P < 0.01 compared with the diabetes group.

 The diameter of retinal vessels in the diabetes group was increased significantly compared with the control group (*P* < 0.01). Compared with the diabetes group, the diameter of the retinal vessels of rats treated with ginsenoside Rb1 (20, 40 mg/kg) was reduced (*P* < 0.05, *P* < 0.01; Fig. 2). 

###  Effect of ginsenoside Rb1 on extravasation of evans blue dye (EBD) 

**Figure 3:**
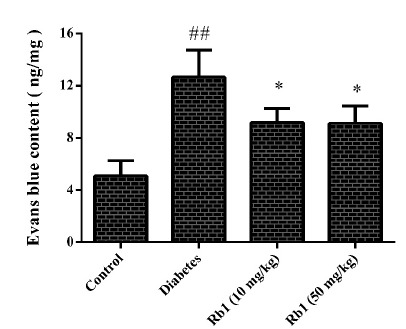
Effect of ginsenoside Rb1 on extravasation of Evans Blue dye. Data are the mean ± SD, (n = 5). ## P < 0.01 compared with the control group; *P < 0.05 compared with the diabetes group.

 The effect of ginsenoside Rb1 on retinal vascular permeability was evaluated by EBD extravasation (Fig. 3). A significant increase in EBD extravasation was observed in the diabetes group (*P* < 0.01). Compared with the diabetes group, treatment with ginsenoside Rb1 (20, 40 mg/kg) decreased EBD extravasation in the retinal vessels of diabetic rats (*P* < 0.05).

###  Effect of ginsenoside Rb1 on histopathologic changes 

**Figure 4:**
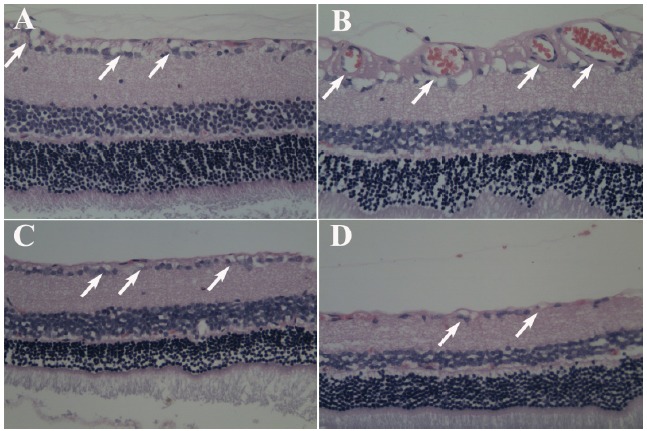
Effect of ginsenoside Rb1 on histopathologic changes (H&E). **A**: Control group; **B**: Diabetes group; **C**: Ginsenoside Rb1 (20 mg/kg) group; **D**: Ginsenoside Rb1 (40 mg/kg) group. Magnification (× 400).

 The histopathologic changes of retinal vessels were investigated in diabetic rats treated or not treated with ginsenoside Rb1 (20, 40 mg/kg). There was a significant increase in the diameter of retinal vessels in rats of the diabetes group (arrows in Fig. 4). However, ginsenoside Rb1 (20, 40 mg/kg) decreased the diabetes-induced increase in the diameter of retinal vessels.

###  Effect of ginsenoside Rb1 on malondialdehyde (MDA) content 

**Figure 5:**
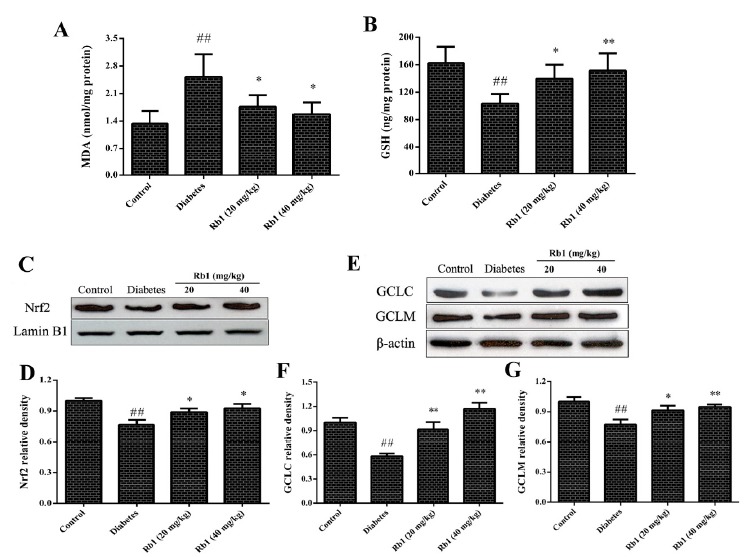
Effect of ginsenoside Rb1 on MDA content (**A**), GSH level (**B**), Nrf2 content (**C**: Representative photographs of Nrf2 in western blots; **D**: Quantitative analyses of Nrf2 content) and the expression of GCLC and GCLM (**E**: Representative photographs of GCLC and GCLM in western blots; **F** and **G**: Quantitative analyses of GCLC and GCLM). Data are the mean ± SD, (n = 3 or 5). ##P < 0.01 compared with the control group; *P < 0.05, **P < 0.01 compared with the diabetes group.

 MDA content was increased significantly in the retinas of diabetic rats (2.52±0.57 nmol/mg protein) compared with the control group (1.33±0.32 nmol/mg protein) (*P* < 0.01). Compared with the diabetes group, the MDA content after treatment with ginsenoside (20, 40 mg/kg) was reduced (1.76±0.29 and 1.57±0.30 nmol/mg protein, respectively, *P* < 0.05; Fig. 5A).

###  Effect of ginsenoside Rb1 on GSH levels 

 In the control group, the GSH level in the retina was 162.7±23.7 ng/mg protein. There was a significant decrease in the GSH level (103.5±14.1 ng/mg protein) in the diabetes group (*P* < 0.01) as compared with the control group. Compared with the diabetes group, treatment with ginsenoside Rb1 (20, 40 mg/kg) resulted in a significant increase in the GSH level (139.5±20.8 and 151.4±25.1 ng/mg protein, respectively) in the retina (*P* < 0.05, *P* < 0.01, respectively; Fig. 5B). 

###  Effect of ginsenoside Rb1 on Nrf2 content 

 Nrf2 content in the nuclei of retinal cells was assayed by western blotting. Compared with the control group, Nrf2 content was decreased in the diabetes group (*P* < 0.01). Treatment with ginsenoside Rb1 (20, 40 mg/kg) augmented Nrf2 content in the nuclei of retinal cells (*P* < 0.05; Fig. 5 C, D). 

###  Effect of ginsenoside Rb1 on expression of glutathione cysteine ligase catalytic subunit (GCLC), and glutathione cysteine ligase modulatory subunit (GCLM) 

 Expression of GCLC and GCLM in the diabetes group decreased markedly as compared with that in the control group (*P* < 0.01). However, after treatment with ginsenoside Re (20, 40 mg/kg), expression of GCLC and GCLM increased significantly (*P* < 0.05 and *P* < 0.01, respectively; Fig. 5 E-G). 

## Discussion

 There are studies which indicated a protective role for alcoholic *ginseng* root extract and Korean red *ginseng* powder agonist DR^16^ ^,^ ^17^. However, the alcoholic extract and power of *ginseng* contain many compounds. Therefore, it is still unclear which compound of *ginseng* has the property of preventing DR. The present findings suggest that ginsenoside Rb1 abrogated (at least in part) the diabetes-induced increase in the diameter of and histopathologic changes in retinal vessels. Ginsenoside Rb1 also reduced the permeability of retinal blood vessels in diabetic rats. Ginsenoside Rb1 augmented levels of Nrf2 and GSH and, therefore, attenuated oxidative-stress injury to the retina. Thus, this work constitutes the ﬁrst study demonstrating that ginsenoside Rb1 can attenuate DR by regulating the antioxidative function in STZ-induced diabetic rats.

 Diabetes mellitus predominantly affects the microvascular circulation of the retina to result in a range of structural changes. Ultimately, these changes lead to altered permeability, hyperproliferation of endothelial cells, edema, and abnormal vascularization of the retina with the resulting loss of vision. Dilation of retinal arterioles has been observed in the early stages of DR^18^. This can be assumed to lead to hyperperfusion so that the arterial blood pressure is transmitted to the capillary bed, where it contributes to the formation of microaneurysms, hemorrhage, and breakdown of the blood-retina barrier. The vascular unit of the retina is composed of endothelial cells, astrocytes and pericytes. The latter have a crucial role in maintenance of vascular stability, and early depletion of pericytes is a hallmark of DR. Pericyte loss increases the proliferation of endothelial cells, thereby contributing to microaneurysm formation in retinal vessels^19^. Pericyte dysfunction also leads to capillary dilation, microaneurysms and increased vascular permeability, resulting in vascular leakage and macular edema^20^. 

 The present study showed that diabetes mellitus led to a significant increase in the diameter of retinal vessels and EBD extravasation. Consistent with the results of fundus photography, histopathology showed that diabetes mellitus caused an increase in the diameter of retinal vessels. However, treatment with ginsenoside Rb1 decreased the diameter and permeability of retinal blood vessels. Ginsenoside Rb1 also attenuated the pathologic changes induced by hyperglycemia. These findings suggest that ginsenoside Rb1 can attenuate DR in STZ-induced diabetic rats.

 Chronically increased glucose levels have a role in the impairment of cellular repair mechanisms. Hyperglycemia contributes to DR development. The present study showed that treatment with ginsenoside Rb1 had no effect on blood glucose levels. Hence, it is reasonable to conclude that the ameliorative effect of ginsenoside Rb1 on DR in STZ-induced diabetic rats was not related to a reduction in blood glucose levels. 

 In diabetes mellitus, the retinal antioxidant defense system is compromised. GSH levels are decreased and those of its oxidized form are increased^21^. GSH is a principal low-molecular-weight thiol antioxidant. The synthesis of GSH from its constituent amino acids involves the actions of two enzymes: glutamate-cysteine ligase (GCL) and GSH synthetase. GCL is the rate-controlling enzyme in the pathway of GSH synthesis, and is a heterodimer composed of GCLC and GCLM. The latter modulates the catalytic properties of GCLC by lowering its sensitivity to the inhibition of GSH and by increasing its affinity to glutamate. Without the presence of GCLM, GCLC would exert its function poorly *in vivo* ^22^. 

 In addition to cellular and enzymatic antioxidant defense systems, the cell is also equipped with Nrf2, which is a transcription factor activated by oxidants to regulate genes containing antioxidant response element (ARE)^23^. The basal and inducible expression of GCLC and GCLM is mediated by ARE. The latter is an enhancer sequence that regulates, at the transcriptional level, antioxidant enzymes, which are crucial for maintaining cellular redox status and protection against oxidative damage^24^. It has been shown that Nrf2 is the principal transcription factor that regulates ARE-mediated gene transcription^25^. During oxidative stress, Nrf2 is translocated to the nucleus to upregulate expression of the genes involved in antioxidant defense. Nrf2 is the main transcription factor required for the activation of *GCLC* and *GCLM* in humans *via* binding with ARE^26^. 

## Conclusions 

 In the present study, we observed that treatment with ginsenoside Rb1 resulted in an increase in nuclear translocation of Nrf2 in the retinas of STZ-induced diabetic rats. Moreover, our data showed that expression of GCLC and GCLM was enhanced after treatment with ginsenoside Rb1. In accordance with these findings, ginsenoside Rb1 also led to an increase in GSH content followed by the decrease in the MDA level. These results suggest that the protective effects of ginsenoside Rb1 can be attributed (at least in part) to anti-oxidative properties due to augmentation of Nrf2-induced expression of GCLC and GCLM. The current study suggests that ginsenoside Rb1 can attenuate DR by regulating the antioxidative function in rat retinas. 
